# DNA hydroxymethylation differences underlie phenotypic divergence of somatic growth in Nile tilapia reared in common garden

**DOI:** 10.1080/15592294.2023.2282323

**Published:** 2023-11-27

**Authors:** Ioannis Konstantinidis, Pål Sætrom, S.O. Brieuc, Kjetill S. Jakobsen, Hannes Liedtke, Caroline Pohlmann, Thomais Tsoulia, Jorge M. O. Fernandes

**Affiliations:** a Faculty of Biosciences and Aquaculture, Nord University, Bodø, Norway; b Department of Clinical and Molecular Medicine, Norwegian University of Science and Technology, Trondheim, Norway; c Department of Computer Science, Norwegian University of Science and Technology, Trondheim, Norway; d Bioinformatics core facility-BioCore, Norwegian University of Science and Technology, Trondheim, Norway; e K.G. Jebsen Center for Genetic Epidemiology, Norwegian University of Science and Technology, Trondheim, Norway; f Center for Ecological and Evolutionary Synthesis (CEES), Department of Biosciences, University of Oslo, Oslo, Norway

**Keywords:** Epigenetics, DNA hydroxymethylation, domestication, somatic growth, teleosts, phenotypic plasticity

## Abstract

Phenotypic plasticity of metabolism and growth are essential for adaptation to new environmental conditions, such as those experienced during domestication. Epigenetic regulation plays a key role in this process but the underlying mechanisms are poorly understood, especially in the case of hydroxymethylation. Using reduced representation 5-hydroxymethylcytosine profiling, we compared the liver hydroxymethylomes in full-sib Nile tilapia with distinct growth rates (3.8-fold difference) and demonstrated that DNA hydroxymethylation is strongly associated with phenotypic divergence of somatic growth during the early stages of domestication. The 2677 differentially hydroxymethylated cytosines between fast- and slow-growing fish were enriched within gene bodies (79%), indicating a pertinent role in transcriptional regulation. Moreover, they were found in genes involved in biological processes related to skeletal system and muscle structure development, and there was a positive association between somatic growth and 5hmC levels in genes coding for growth factors, kinases and receptors linked to myogenesis. Single nucleotide polymorphism analysis revealed no genetic differentiation between fast- and slow-growing fish. In addition to unveiling a new link between DNA hydroxymethylation and epigenetic regulation of growth in fish during the initial stages of domestication, this study suggests that epimarkers may be applied in selective breeding programmes for superior phenotypes.

## GRAPHICAL ABSTRACT

**Figure:**
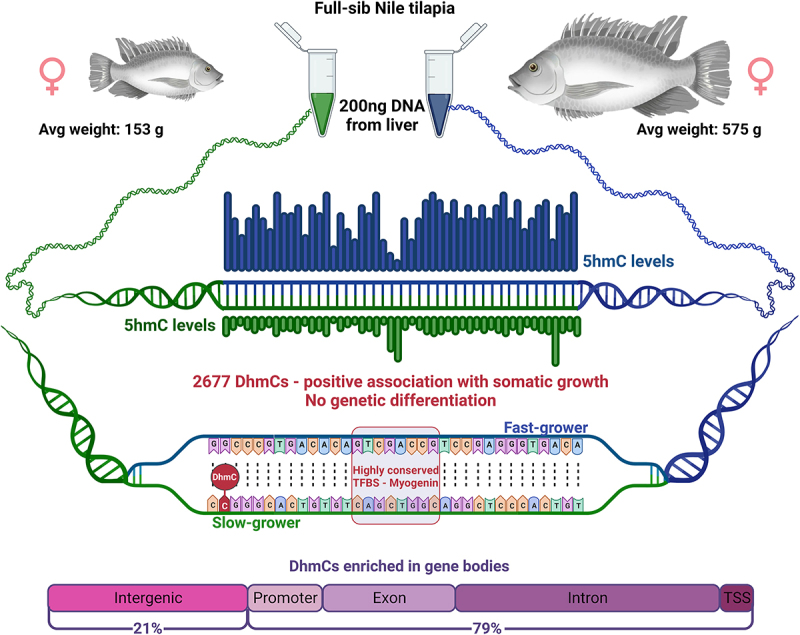

## Introduction

The genetic screening and selection of desirable traits lead to the optimization of animal breeding and farming both for terrestrial and aquatic species (see review [1]). However, phenotypic characteristics are not determined solely by the DNA sequence itself. Within the past decade, there has been increasing interest in epigenetic trait selection and improvement of farmed plants and animals [2–5]. These efforts are being pursued in several terrestrial species, such as cattle, pig, goat, sheep and poultry, and target primarily animal welfare, health, and productivity [6–9]. In aquaculture, knowledge on the epigenetic regulation of economically valuable traits is scarce but there are several studies showcasing the importance of these modifications in fish, including sexual maturation, sex determination and differentiation, growth, nutrition, health, and stress [10].

Domestication of wild animals is often associated with early and rapid molecular adaptations that are reflected in changes in behaviour and phenotype [11–13]. Phenotypic plasticity and epigenetic changes rather than mutations across several individuals are the more likely explanations for the simultaneous emergence of distinct phenotypes within a single generation of domestication [14].

Somatic growth is one of the most desirable traits for domesticates intended for food production, including aquaculture species [15,16]. Improved growth is directly associated with the process of domestication due to the optimal conditions provided by humans such as temperature, nutrition, feeding to saturation, as well as protection from predators and pathogens. These environmental factors shape drastically the epigenome, thus altering the phenotypic variability within the population.

Understanding the epigenetic interactions that promote phenotypic divergence of somatic growth during the first stages of domestication could provide valuable clues for the improvement of breeding management, trait selection, and sustainable production.

In human, liver DNA methylation has been identified as a key epigenetic regulator involved in gene transcription, liver and insulin metabolism [17] as well as insulin resistance [18] which are highly relevant in nutrient uptake, anabolic actions, and consequently somatic growth. 5-hydroxymethylcytosine (5hmC) is an oxidized derivative of 5-methylcytosine (5mC). Its oxidation occurs through ten-eleven translocation (TET) activity [19] and 5hmC has been previously acknowledged as a stable epigenetic modification [20]. Recent studies have shown that TET enzymes play a critical role in DNA demethylation and are responsible for the regulation of genes such as the peroxisome proliferator-activated receptor gamma coactivator 1-alpha (PPARGC1A) [21,22], which is associated with metabolic reprogramming in liver and the coordination of genes involved in glucose and fatty acid metabolism [23]. Also, enriched 5-hydroxymethylcytosine (5hmC) within gene bodies has been associated with H3K4me2/3 histone marks [24] and increased levels of gene expression [25]. DNA hydroxymethylation in liver and its association with metabolism and somatic growth has so far not been investigated in fish, with the exception of one sister publication from our group [26]. Investigating the impact of DNA hydroxymethylation in the liver, a tissue that is strongly associated with the regulation of metabolism and growth in teleosts, will likely provide evidence for epigenetic-mediated phenotypic plasticity for somatic growth.

Here, we provide the liver hydroxymethylome maps at single nucleotide resolution in fast- and slow-growing wild Nile tilapia (*Oreochromis niloticus*) reared in common garden and undergoing domestication. We were also able to associate our results with genetic variants among the two distinct phenotypes because the approach used (reduced representation 5-hydroxymethylcytosine profiling) does not involve bisulphite conversion. Genes involved in metabolism and growth-related pathways were greatly enriched in differentially hydroxymethylated cytosines (DhmCs) between fast- and slow-growing fish. On the other hand, single nucleotide polymorphism (SNP) analysis revealed no genetic differentiation between the two groups, suggesting that hydroxymethylation is strongly associated with phenotypic divergence in early stages of domestication and possibly gene-specific accelerated evolution.

## Results

### Characterization of RRHP libraries from fish with distinct growth phenotypes

**Figure 1.**
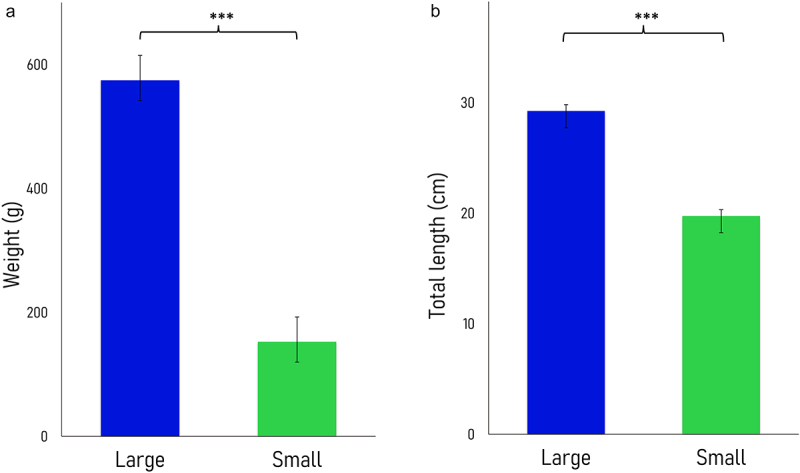
Barplots displaying the average weight (A) and length (B) of Nile tilapia females between the large (fast-growing, dark blue) and small phenotypes (slow-growing, green). Asterisks reflect significant differences for both weight and length between fish with distinct growth rates (one-tailed t-test, p-value <0.0001). Data are represented as means ± standard deviations (*n* = 5).

Fertilized eggs (G1) were collected from a Nile tilapia male and female pair (G0) that spawned for the first time in captivity. After 5 months, significant differences in size and weight were observed within G1 full siblings reared in common garden (Figure 1). Liver samples were obtained from five fast-growing (i.e., large) and five slow-growing (i.e., small) females (*n* = 5). The large fish were 3.8-fold heavier and 1.5-fold longer than their small counterparts. The average weight was 574.6 ± 40.1 g and 152.6 ± 32.8 g and the average length was 29.2 ± 0.6 cm and 19.7 ± 1.5 cm in the fast- and slow-growing fish groups, respectively. Due to the nature of the reduced representation hydroxymethylcytosine profiling (RRHP) protocol (see Methods), libraries from liver samples with higher levels of 5hmCs produced a higher number of sequencing reads. On average, 52.5 M and 18.5 M raw reads were produced from the fast- and slow-growing groups, respectively (*n* = 5). Out of these, an average of 31.2 M and 10.1 M reads were uniquely mapped to the reference genome [27] with an average 58.5% alignment efficiency (Supplemental Fig. S1; Supplemental Table S1A). There was a positive correlation between 5hmC enrichment and weight (Supplemental Table S1B) as well as length (Supplemental Table S1C; Supplemental Fig. S2).

### Genome-wide hydroxymethylation in liver of slow- and fast-growing nile tilapia

**Figure 2.**
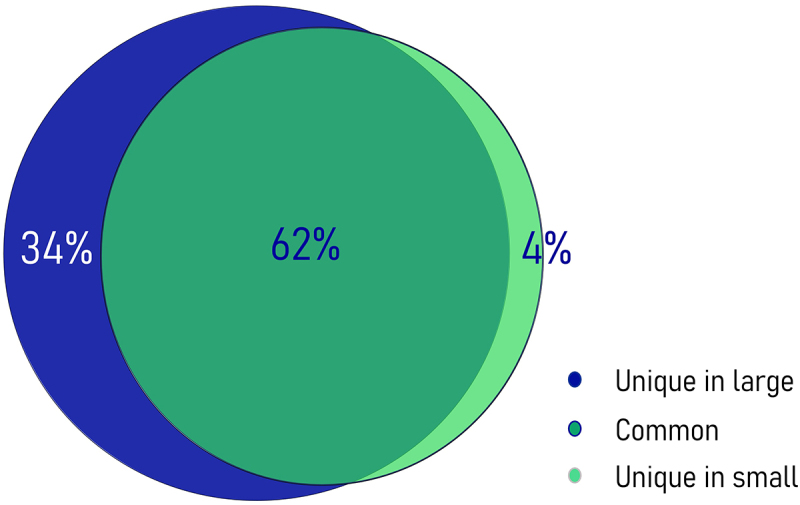
Venn diagram showing the percentages of unique and common 5hmC sites. Hydroxymethylated cytosines identified exclusively within the large (i.e., fast-growing) (34%) and small (i.e., slow-growing) (4%) fish are represented in dark blue and light green, respectively, while common hydroxymethylated sites (62%) are shown in dark green. The 5hmC enrichment was obtained from 291,847 CCGG sites with non-zero values among all samples (*n* = 5).

**Figure 3.**
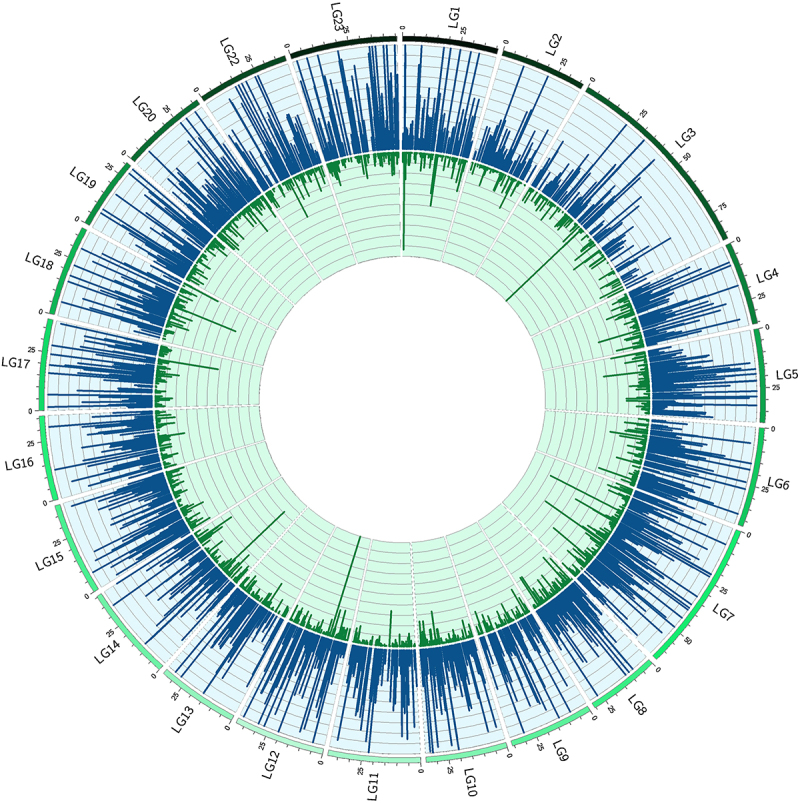
Circular representation of the Nile tilapia nuclear genome with each arc corresponding to a linkage group (LG1-LG23). Blue (oriented out) and green (oriented in) peaks represent hyper-DhmCs in the fast- and slow-growing groups, respectively. The enrichment of single DhmCs is based on the average filtered counts (*n* = 5) and is reflected on the height of each peak.

**Figure 4.**
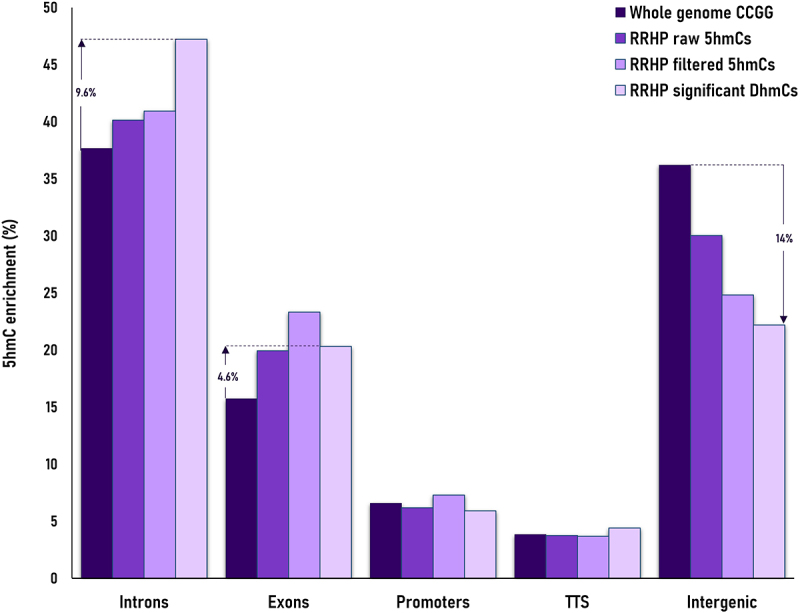
Barplot displaying the comparison of 5hmC enrichment per annotated feature (introns, exons, promoters, transcription termination sites -TTS and intergenic regions) between potential RRHP targets (whole genome CCGG sites; dark purple), RRHP raw 5hmC sites (purple), RRHP filtered sites (light purple) and RRHP differentially hydroxymethylated cytosines between fast- and slow-growing phenotypes (very light purple).

Hydroxymethylation was present across the whole genome, albeit at relatively low levels in both phenotype groups. Most 5hmCs with non-zero counts across all samples, were present in both groups (62%). However, an 8-fold enrichment of unique 5hmC sites in the group of fast-growing fish reflected the increased hydroxymethylation levels for this phenotype (Figure 2). In total, we identified 1,096,820 potentially hydroxymethylated sites; however, after filtering only 138,000 CCGG sites were considered to be substantially hydroxymethylated. Their comparison revealed 2,677 5hmCs that were differentially hydroxymethylated (DhmCs, |log_2_(fold change (FC) in DNA hydroxymethylation levels) >1; q < 0.05) between the two groups, of which 2,237 were hyperhydroxymethylated (log_2_FC >1) and 440 were hypohydroxymethylated (log_2_FC <1) in fast-growing fish compared to their slow-growing counterparts (Figure 3). DhmCs were mostly enriched within gene bodies including promoters (79%) compared to intergenic regions (21%). There was a relative enrichment of 9.6% within introns and 4.6% within exons as well as a 14% depletion within intergenic regions compared to all genomic CCGG sites (Figure 4).

### DhmCs are associated with intracellular signal transduction, development, and growth

Genes containing hyper-DhmCs in the group of fast-growing fish were associated primarily with gene ontology (GO) terms such as intracellular, small GTPase, Rho protein and Ras protein signal transductions, as well as with growth, developmental growth, skeletal system development, muscle structure development, and striated muscle tissue development (q < 0.01; Supplemental Table S2A). Regarding the hypohydroxymethylated genes in the same group, GOs were associated with cell adhesion, axon and neuron projection extension, and axonogenesis (q < 0.01; Supplemental Table S2B). Likewise, functional enrichment analysis using our modified gene list and human orthologues (see Methods; Supplemental Tables S3A and S3B) revealed eight enriched KEGG pathways. Hypo- and hyper-hydroxymethylated genes in fast-growing fish were significantly enriched for the axon guidance and the extracellular matrix (ECM)-receptor interaction pathways (q < 0.05; Supplemental Table S2C and S2D). Additionally, genes containing hyper-DhmCs were enriched for the PI3K-Akt signalling pathway, which is a fundamental pathway of cell cycle, proliferation, metabolism, and growth [28], as well as for the Rap1 signalling and focal adhesion pathways, which are involved in cell–matrix interactions [29,30], MAP kinase activity, cell cycle, and cell survival [31,32]. Another interesting finding was the significantly enriched pathway of protein digestion and absorption, which is related to liver metabolism [33] (q < 0.01; Supplemental Table S2D).

Several genes were associated with the above GO terms and linked to growth (q < 0.01; Supplemental Table S4). Most growth-related genes (72 out of 81) were found to be hyperhydroxymethylated in fast-growing individuals, indicating a positive association between 5hmC levels and growth. Of the 102 DhmCs found within these genes, 80 were located within gene bodies and were particularly enriched within introns (Supplemental Fig. S3). The remaining 22 DhmCs were located in intergenic regions and were assigned to genes based on their proximal distance to transcription start sites (TSS). The average proximal distance was calculated at 15,318 bp, with the closest intergenic-DhmC being at 1,246 bp upstream of the fibroblast growth factor 13 (*fgf13*) TSS, and the farthest at 84,085 bp upstream of the retinoid X receptor alpha (*rxra*) TSS. Among the genes with the highest enrichment in DhmCs were the zinc finger protein FOG family member 2 (*zfpm2*; 4 DhmCs), which forms complexes with transcription factors such as the GATA4. In the liver, these complexes have functions related to lipid metabolism, glycolysis/gluconeogenesis, and phase 1 functionalization of compounds [34,35]. Additionally, we identified three DhmCs within the membrane associated guanylate kinase WW and PDZ domain containing 2 (*magi2*), which suppresses AKT activity by enhancing PTEN function [36], and 3 DhmCs within the fibroblast growth factor 1 (*fgf1*) which is involved in the regulation of MAPK activity and fibroblast growth factor receptor signalling [37,38].

**Figure 5.**
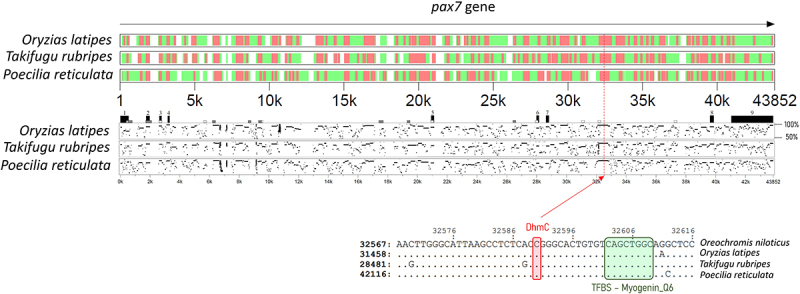
Visual representation of conserved regions of *pax7* among four fish species. Highly conserved, intermediately conserved and non-conserved regions of the gene are represented by red, green and white colour, respectively. Numbered black boxes represent the exons of *pax7* in Nile tilapia while each dot-plot corresponds to the pairwise alignments (50–100% similarity) between Nile tilapia and the fish species that is designated on the left side. The zoom-in below shows the exact position of the DhmC among fast- and slow-growing Nile tilapia as well as the myogenin TFBS sequence that is highly conserved among these four species.

Hyper-DhmCs in fast-growing fish were detected within several paired box family genes (*pax3*, *pax5*, *pax7*) as well as the pax3 and pax7 binding protein 1 (*paxbp1*). Interestingly, a single DhmC within the 7th intron of *pax7* was located in a highly conserved region (334 bp long) among four fish species including Nile tilapia, medaka (*Oryzias latipes*), Japanese puffer (*Takifugu rubripes*) and guppy (*Poecilia reticulata*). To better understand the significance of this conserved non-coding DNA sequence within *pax7*, we searched for cis-regulatory elements (CREs) and transcription factor binding sites (TFBSs). Strikingly, we identified a myogenin transcription factor binding site (TFBS_Myogenin_Q6) in close proximity to the DhmC (11 bp downstream; Figure 5).

### Single nucleotide polymorphisms (SNPs) were not significantly different between the two groups with distinct growth rates

Single nucleotide polymorphism analysis revealed 140,932 SNPs, of which 66,259 SNPs were considered valid for the estimation of genetic difference between groups. Among these, 18,143 and 12,131 SNPs were polymorphic exclusively in fast- and slow-growing fish, respectively, while the other 35,985 SNPs were polymorphic in both groups (Supplemental Table S5A). After filtering for missing values and biallelic loci 30,982 SNPs were tested for significant differences between groups. In total, 86 SNPs had a significant association with size (Supplemental Table S5B; *p* < 0.01) but after FDR correction (q < 0.05) no loci were associated with size differences between the two fish groups.

## Discussion

Genes containing differentially hydroxymethylated cytosines (DhmCs) could explain in part the observed phenotypic differences between the fast- and slow-growing, full-sib Nile tilapia. In particular, several growth factors (*fgf1*, *fgf10,* and *fgf13*), kinases (*eif2ak4*, *stk40*, *akap13*, *akap6*, *akt1*, *magi2*, *prkcq,* and *pkdcc*) and receptors (*rgma*, *rxra, bmpr2*, *dcc*, *il17rb,* and *pparg*) were found to be hyperhydroxymethylated in fast-growing fish and displayed a positive association between 5hmC levels and growth. While we cannot exclude the possibility that the improved growth rate was due to higher food consumption, there were no differences in 5hmC levels within *npy*, *cck,* or *lepr*/*lepa* genes, which are directly associated with appetite and feeding behaviour in fish.

An interesting finding was the enrichment of DhmCs within biological processes related to skeletal system and muscle structure development. It is well known that the neuroendocrine system meticulously controls the function and hormone secretion of the pituitary gland as well as the organs responsible for energy absorption and storage, namely, the liver, the adipose tissues, and the skeletal muscle [39]. The interplay and exchange of molecules among those tissues, for example the process of gluconeogenesis in the liver which occurs due to the delivery of alanine from skeletal muscle [40], result in a highly sophisticated biological network that is regulating metabolic processes and ultimately growth. Among the genes linked to skeletal system development, we identified the lysophosphatidic acid receptor 1 (*lpar1*), which is a lipid mediator involved in the regulation of intracellular-free calcium concentration [41] and proliferation [42], the serine/threonine-protein kinase N2 (*pkn2*) and PR domain zinc finger protein 5 (*prdm5*) involved in the regulation of mitotic cell cycle [43,44], and the extracellular tyrosine-protein kinase (*pkdcc*), which is part of the secretory pathway, mediator of extracellular protein phosphorylation, chondrocyte differentiation, and bone mineralization [45]. The liver plays an important role in osteoclastogenesis by IGFBP1-mediated bone resorption [46]. Interestingly, *pkdcc* is strongly expressed in mice liver [47] and its suppression (either by homozygous knockout or biallelic disruption) results in skeletal abnormalities in both mice [47] and humans [48]. Previous studies have shown that muscle growth stimulates bone mass [49] and similar growth and differentiation factors are involved in muscle and bone diseases such as sarcopenia and osteoporosis, respectively [50]. Considering the close relationship of bone and muscle cells, and the role of *pkdcc* in skeletal development, it is plausible that could be epigenetically regulated in response to environmental cues and play a role in the phenotypic divergence that we observed in Nile tilapia.

The liver is also the main source of insulin-like growth factor I (*igf-I*) production and plays a key role in somatic growth. In human, DNA methylation has been recently linked to liver and insulin metabolism [17] as well as insulin resistance [18]. In particular, the expression of the peroxisome proliferator-activated receptor gamma coactivator 1-alpha (PPARGC1A) [18,22] as well as its epigenetic regulation by the deacetylase Sirt1 and acetyltranferase GCN5 [23] have been linked to glucose and fatty acid metabolism. In this study, we identified hyper-DhmCs in the fast-growing individuals within the first intron of *ppargc1a* and the fourth intron of the peroxisome proliferator-activated receptor gamma (*pparg*) (Supplemental Table S3). Active DNA demethylation mechanisms that promote 5hmC enrichment are potentially an indicator of epigenetic control of the *ppargc1a* in the liver that could result in increased insulin metabolism and somatic growth.

Among the most enriched biological processes, we identified several terms related to intracellular signalling (GO:0035556) such as small GTPase-mediated, Rho and Ras protein signal transduction (GO:000–7264/7266/7265; q < 0.01). The Ras signal transduction pathway is a fundamental downstream regulator of cell growth through the regulation of cellular responses to growth factors and their binding to cell surface receptors [51]. The differentially hydroxymethylated genes included several Rho and Rac/Cdc42 guanine nucleotide exchange factors (*arhgef17*, LOC100708472: *arhgef12*, *arhgef37*, *arhgef10l*, LOC100705949: *arhgef25*, *arhgef4,* and *arhgef6*), brefeldin A-inhibited guanine nucleotide exchange proteins (*arfgef1* and LOC100708121: *arfgef2*), as well as the Rho GTPase-activating protein 7 (LOC100696392: *dlc1*) and the FYVE RhoGEF and PH domain containing proteins 1 and 3 (*fgd1* and LOC100689713: *fgd3*), which are involved in cell growth, cell survival, and gene transcription. The functional enrichment analysis of our modified gene list unveiled fundamental biological pathways that are both structurally and functionally crucial in cell signalling, adhesion, motility, migration, differentiation, proliferation, and apoptosis. The highest enrichment was observed in focal adhesion and ECM-receptor interaction pathways (q < 0.001). Several important ECM-related proteins were found to be differentially hydroxymethylated. In particular, from the family of fibronectins, we identified the fibronectin type III domain-containing 5 (*fndc5*) gene that regulates the connective tissue growth factor (CTGF) and transforming growth factor-β (TGF-β), which is involved in deposition of the ECM [52]. Differentially hydroxymethylated genes from the family of laminins comprised the glycoproteins laminin subunit alpha 4 and 5 (*lama4* and *lama5*) as well as the predicted proteins (LOC100707779: *lamb1*, LOC100706244: *lamb2*, LOC109194199: *lamb3*, LOC102077756: *lamc3*) involved in cell migration, proliferation, and adhesion [53]. Additionally, we identified 22 DhmCs within several types of collagen chains (*col14a1, col16a1, col18a1, col19a1, col1a1, col1a2, col24a1, col27a1, col4a1, col4a2, col4a6, col5a1, col5a2, col6a2, col9a1,* and *col9a2*), a major structural component of the ECM. As transmembrane adhesion receptors, integrins facilitate the communication of the extracellular microenvironment with the cytoskeleton [54]. Their importance in intracellular signal transduction makes them essential in several biological functions and processes. Among others, cell growth and proliferation are highly dependent on integrin-anchoring with the ECM and growth factor receptors [55]. Remarkably, 65 differentially hydroxymethylated genes were associated with the PI3K-Akt signalling pathway, which is an important and highly conserved pathway throughout evolution, involved in cell cycle, metabolism, survival, and proliferation [28]. We have reported the presence of differentially methylated CpGs within the *akt3* and *map3k5* genes in association to Nile tilapia growth phenotypes [56], which suggest epigenetic regulation of the PI3K-Akt signalling pathway. Here, we demonstrated that several genes (i.e., *akt1*, *ppp2r5c*, *ppp2ca*, *rptor*, *magi2*, *pdgfrb*, *creb5*, *fgf10*, *insr,* and *jak2*) that play crucial role within the PI3K-Akt signalling pathway were differentially hydroxymethylated between the fast- and slow-growing phenotypes. Furthermore, most DhmCs were intragenic, supporting their functional role in gene transcription. Considering the importance of hydroxymethylation within gene bodies and the higher enrichment of DhmCs within the large group of fish, we hypothesize that divergent growth phenotypes can emerge through the epigenetic regulation of metabolic pathways such as PI3K-Akt.

Besides hydroxymethylation mapping at single nucleotide resolution, RRHP provides genome-wide data for the identification of SNPs [57]. In total, we identified 86 significantly different SNPs and 67 haplotypes (more than one SNP per sequencing read) between the two phenotypes (*p* < 0.01). However, after FDR correction neither the SNPs, nor the haplotypes could be associated with size. These results unveil an expected outcome due to the relationship of the two groups (full-sibs), but we cannot entirely dismiss the possibility of genetic variation due to the limitations of the RRHP protocol compared to specific NGS library preparation protocols in genome-wide association studies. Another limitation regarding the identification of significant SNPs among groups is the sample size, which decreases the statistical power of the analysis.

The discovery of CREs within the annotated features of the Nile tilapia genome can improve our understanding of how single-nucleotide substitutions and epigenetic modifications can play a critical role in divergent phenotypes and processes affecting genome adaptation and evolution [58]. We have identified a single DhmC within the 7^th^ intron of *pax7*, which was in close proximity to a *myogenin* TFBS. Considering the evident enrichment of 5hmCs within gene bodies and their roles in gene regulation [59,60] it would be interesting to investigate the impact of this mark on *pax7* expression, a gene that is closely associated with satellite cell activation, proliferation and muscle fibre formation and growth [61,62].

Animal domestication is inextricably linked to genetic variation, environmental factors and their interactions. However, during the early stages of domestication, rapid gene expression changes [11,13] and epigenetic remodelling [12,13] are most likely attributed to epigenetic changes or epimutations as opposed to genetic mutations [63]. Some changes are initiated early in development, but persist to adulthood and can be transmitted across generations [64,65]. With that being said, one must consider the possibility of epigenetic-mediated phenotypic plasticity leading evolution, rather than being reflected by differences in allele frequencies and mutations across several generations [66]. Understanding the importance of the epigenome in early adaptation could provide valuable clues on the evolutionary consequences of epigenetic inheritance during domestication.

Aquaculture offers an outstanding opportunity for the investigation of artificial selection for desirable traits and fast-paced genome evolution. Compared to terrestrial species that have been domesticated for thousands of years, wild populations of fish species such as Nile tilapia (*Oreochromis niloticus*) can be found within their historical native range, thus enabling the study of their earliest molecular responses to domestication. Among the epigenetic mechanisms involved in early adaptation to new environments, DNA hydroxymethylation is a stable epigenetic modification [20] that serves multiple roles in gene regulation [67,68] across various biological contexts [59,69,70] and developmental stages [71]. In regard to evolutionary epigenetics, this DNA modification has received very little attention, even though 5-hydroxymethylcytosines (5hmCs) have higher C to G transversion rate than 5-methylcytosines (5mCs) and unmodified cytosines [72]. Consequently, the establishment of genome-wide 5hmC epimutations can have major implications in gene-specific evolution that reflect phenotypic variation, even as single-5hmC sites within gene bodies and cis-regulatory elements.

## Conclusions

Taken together, the results of this study demonstrate that the epigenetic regulation of the genome in wild animals placed in captivity conditions is potentially one of the first molecular mechanisms towards adaptation during domestication. DNA hydroxymethylation is possibly involved in gene regulation and contributes to phenotypic divergence and functionally targeted evolution. Confirming the biological significance of such modifications and validating epigenetic biomarkers will not only shed light into the molecular mechanisms underlying phenotypic plasticity and animal domestication, but also improve current selective breeding practices, trait enhancement, and thus sustainability in aquaculture.

## Materials and Methods

### Breeding and sample collection

Fertilized Nile tilapia eggs were obtained from wild females caught in the Nile river (Egypt) within a 500 m radius from the following GPS coordinates: latitude 25.6655 and longitude 32.6186. Nine days post hatching, the larvae (G0 generation) were transported to Nord University’s research station (Norway), where they were reared for 8 months in a recirculating aquaculture system (RAS; temperature = 28°C, 13:11 h light:dark cycle, pH = 7.6, oxygen saturation: 90–100%). For breeding, single males were placed together with two females in 250 L aquaria. The aquaria were connected to the RAS, the water had a constant flow of approximately 2 L/min and additional air was provided by air pumps. Females were visually inspected for fertilized eggs in their mouths οn a daily basis. The hatched fry (G1 generation) were collected from one of the two mouthbrooding female after 5–6 days post fertilization (dpf) and were placed for a week in an egg-rocker. From 11 dpf to 2 months of age, the juveniles were kept in a 300 L tank, and afterwards, they were moved to a 1500 L tank. The fish (G1) were full siblings (i.e., originating from a single spawning event involving a single dam and a single sire). They were fed *ad libitum* with 0.15–0.8 mm Amber Neptun pellets (Skretting, Norway) at a maximum density of approximately 5 kg/m^3^ and large differences in size were observed after a 5-month rearing period. In total, liver tissue from five large (i.e., fast-growing) and five small (slow-growing) full-sib G1 females were sampled visually (*n* = 5). This study focused on females because the liver is involved in vitellogenesis and components of yolk precursors may have intergenerational effects [73]. All animals (10) were sexually mature and had fully developed ovaries (Supplemental Table S1D). Prior to sampling, the fish were starved for 48 hours. On the sampling day, they were randomly caught between 8 and 9 am with the use of a net and euthanatized with clove oil (Sigma Aldrich, USA) overdose. Forty ml of diluted clove oil (99% eugenol, 1:10 in 96% ethanol) were added to 20 L of water in an oxygenated tank; the total time of exposure to the anaesthetic was 3 minutes at 28°C. The liver was carefully dissected and samples were collected from the left lobe near the entry point of the portal vein. They were placed in 2 ml sterile cryogenic vials, snap-frozen in liquid nitrogen and stored at −80°C until further analysis. Every comparison performed between the two groups refers to the ‘large (i.e., fast-growing) group’ *versus* the ‘small (i.e., slow-growing) group.’ Positive and negative log fold change (Log FC) values indicate hyper- and hypo-5hmC levels in fast-growing fish, respectively.

### DNA extraction

DNA was extracted from 10 mg liver using the Quick-DNA miniprep plus kit (Zymo Research, USA), according to manufacturer’s recommendations for solid tissues. DNA concentration was calculated using the DNA broad range assay on a Qubit 3.0 fluorometer (Thermofisher Scientific, USA), its integrity was determined on a TapeStation 2200 (AgilentTechnologies, USA) and its purity on a NanoDrop Spectrophotometer 1000 (Thermofisher Scientific).

### RRHP library preparation and sequencing

Reduced representation hydroxymethylation profiling (RRHP) libraries were prepared according to the manufacturer’s protocol (Zymo Research). Detailed information regarding the preparation of the RRHP libraries can be found in our previous work [13]. Due to the nature of the RRHP protocol, samples with higher levels of hydroxymethylation produce a higher number of fragments and therefore more sequencing reads. An equal volume of each library was sequenced using our in-house NextSeq 500/550 (Illumina, USA) in two High Output v2 flow cells (Illumina, USA), according to the manufacturer’s recommendations. To avoid flow cell biases, the libraries of both groups had a balanced representation across both flow cells. A positive but not 5hmC-specific control library was also included in both runs to test for flow cell effects. This control library was produced by omitting the second MspI digestion and half of the volume was sequenced in both flow cells compared to the experimental libraries. To account for the low complexity of the libraries, 34% PhiX control v3 (Illumina) was added to the library pool prior to sequencing.

### Bioinformatic analyses

RRHP sequencing data were analysed as detailed in Konstantinidis et al. [13,74]. In brief, raw reads were quality checked (Q > 20) and adapter trimmed using trim_galore v0.4.4 (Babraham Bioinformatics) [75]. Alignments were performed using the latest Nile tilapia genome assembly (O_niloticus_UMD_NMBU) [27] and Bowtie v0.12.8 aligner [76]. Chromosome, starting position and strand information were extracted from all the reads that started with CCGG on both strands. This information was then used as input in R [77]. A count matrix was created containing the sum of 5hmC instances per sample and CCGG location. The matrix was filtered twice for low 5hmC signals. Initially, 691,128 5hmC locations were removed when six or more, out of 10 samples, had 0 counts. This filter was applied to more than six samples to ensure that the eliminated CCGG sites had very low counts in both phenotypes. Afterwards, the median of the remaining data was calculated (19) and 267,692 additional 5hmC sites were removed when six or more samples had values below the median threshold. A total of 138,000 substantially enriched 5hmC sites were used for further analysis. Our design considered size differences between the two distinct phenotypes as the primary source for variation. Therefore, the count data were log2 transformed using the function voom and a linear model was fitted using weighted least squares for each site in R package limma. Computation of moderated *t*- and B-statistics of differential hydroxymethylation levels was performed using the function eBayes. The adjusted *p* values were calculated with the Benjamini-Hochberg method. All sites with an adjusted *p* value ≤0.05 were extracted using the topTable function and their annotation was performed using the latest NCBI *Oreochromis niloticus* annotation release 104 and the Perl script ‘annotatePeaks.pl’ in HOMER [78]. Due to the insufficient information (unassigned gene symbols and/or orthology status) of 963 out of the 1 880 genes containing significantly different 5hmC levels, we used their protein names based on NCBI’s eukaryotic genome annotation pipeline (NCBI *Oreochromis niloticus* Annotation Release 104) in combination with the human gene symbols in UniProt Protein knowledgebase and modified our gene list accordingly [Gene Symbols (NCBI-UniProtKB)]; Supplemental Tables S2A and S2B). Functional enrichment analysis for biological processes and KEGG pathways were performed based on the data sources available for Nile tilapia and Human orthologs, respectively, using the online platform g:Profiler [79], version e98_eg45_p14_ce5b097 with Benjamini-Hochberg FDR [80] as our multiple testing correction method and a significance threshold of 0.01.

### Genetic variation analysis using RRHP data for SNP calling

The same adapter and quality trimmed fastq files as described above were used for the identification of SNPs across individuals and among fast- and slow-growing fish groups. All reads were mapped to the reference genome using Bowtie2 [81] in end-to-end mode while retaining only the best alignment for each read. SNPs were identified using Stacks [82] with default parameters while haplotypes were reported when more than one SNP was present in a single read. Loci with missing values were excluded due to 5hmC-specific enrichment bias between groups. The SNP-size and haplotype-size associations were conducted in Plink [83]. Predictions of protein stability after missense mutations were calculated using the online platform I-Mutant 2.0 [84]. For the identification and visualization of conserved regions among species on specific genes of interest, we used the online software MultiPipMaker [85] while searches for common TFBSs were performed using the online tool MULAN in combination with the multiTF algorithm [86].

## Supplementary Material

Supplemental_Table_S5.xlsx

Supplemental_Table_S3.xlsx

Supplemental_Material.pdf

Supplemental_Table_S4.xlsx

## Data Availability

The DNA hydroxymethylation dataset generated in this study has been submitted to the NCBI Sequence Read Archive (SRA) repository, under the accession number PRJNA665120.
