# Supplementary material for: DNA hydroxymethylation differences underlie phenotypic divergence of somatic growth in Nile tilapia reared in common garden
Source: Epigenetics. 2023 Nov 27;18(1):2282323. doi: 10.1080/15592294.2023.2282323 (PMC10732659; doi:10.1080/15592294.2023.2282323)
Supplement: Supplemental_Material.pdf [file KEPI_A_2282323_SM3816.pdf]

Title: DNA hydroxymethylation differences underlie phenotypic divergence of somatic growth in Nile tilapia reared in common garden

Authors

Ioannis Konstantinidis[1], Pål Sætrom[1,2,3,4,5], Marine Servane Ono Brieuc[6], Kjetill Sigurd Jakobsen[6], Hannes Liedtke[1], Caroline Pohlmann[1], Thomais Tsoulia[1] and Jorge M. O. Fernandes[1]*

Affiliations

[1]Faculty of Biosciences and Aquaculture, Nord University, Bodø, Norway

[2]Department of Clinical and Molecular Medicine, Norwegian University of Science and Technology, Trondheim, Norway

[3]Department of Computer Science, Norwegian University of Science and Technology, Trondheim, Norway.

[4]Bioinformatics core facility-BioCore, Norwegian University of Science and Technology, Trondheim, Norway

[5]K.G. Jebsen Center for Genetic Epidemiology, Norwegian University of Science and Technology, Trondheim, Norway

[6]Center for Ecological and Evolutionary Synthesis (CEES), Department of Biosciences, University of Oslo, Oslo, Norway

*Corresponding author: Professor Jorge Fernandes, email: jorge.m.fernandes@nord.no

Supplemental Tables

Supplemental Table S1A.

Next generation sequencing output of both phenotypes (n=5). The raw, remaining after quality control and adapter trimming, uniquely mapped, multiple mapped and failed to map64reads are displayed for each sample.

Samples	Raw reads	Quality & trimming	Unique Mapped	Multi Mapped	Failed to Map
**BL1**	29951172	29925972	17809316	6751993	5364663
**BL2**	64951163	64904447	40785396	13434728	10684323
**BL3**	46520470	46497025	29212772	10468899	6815354
**BL4**	65123992	65114241	33150251	22994436	8969554
**BL5**	56155408	56140069	34910811	12068031	9161227
**SL1**	21105349	21083382	11905746	5500952	3676684
**SL2**	27287570	27273609	15505064	7532191	4236354
**SL3**	7105158	7102657	4127283	1785881	1189493
**SL4**	24225784	24216931	14377932	5611876	4227123
**SL5**	12690867	12686646	7388386	3159084	2139176

Supplemental Table S1B.

Calculation of Pearson correlation coefficient between raw number of RRHP reads and total weight of animals (n=10), R^2, T-statistics and P-value.

NGS raw reads - Total Weight Correlation					
Pearson	R-squared	T-statistic	P-value	n	DF
0.889370956	0.790980698	5.502179318	0.000572272	10	8

Supplemental Table S1C.

Calculation of Pearson correlation coefficient between raw number of RRHP reads and total length of animals (n=10), R^2, T-statistics and P-value.

NGS raw reads - Total Length Correlation					
Pearson	R-squared	T-statistic	P-value	n	DF
0.841747112	0.7085382	4.40997175	0.002256491	10	8

Supplemental Table S1D.

Recorded measurements of total weight (g), total length (cm), standard length (cm), weight of ovaries (g) and gonadosomatic index (GSI). Below, calculations of average weight and length, their standard deviations and Student's t-test revealing the statistical significance of both weight and length between phenotypes. The animals were females, full-sibs and 5 months old. The large and small phenotypes were assigned to the names (BL) and (SL), respectively.

Sampling Label	Sex	Sample	Total Weight (g)	Total Length (cm)	Standard Length (cm)	Ovaries weight (g)	GSI
F1-5mts-1	female	BL1	512	29	24	16.3	3.18
F1-5mts-6	female	BL2	634	30.2	25.5	20.4	3.22
F1-5mts-8	female	BL3	587	29.5	24.5	15.2	2.59
F1-5mts-11	female	BL4	584	28.5	23.5	16.2	2.77
F1-5mts-12	female	BL5	556	29	23.7	18	3.24
F1-5mts-3	female	SL1	191	21.4	17.5	6.8	3.56
F1-5mts-7	female	SL2	113	17.8	14	2.9	2.57
F1-5mts-9	female	SL3	138	18.6	15	4.9	3.55
F1-5mts-13	female	SL4	192	21.6	17.7	5.2	2.71
F1-5mts-14	female	SL5	129	19.3	15.7	3.9	3.02

Supplemental Table S2A. Lists of significantly enriched biological processes (BP) for **(A)** hyper-hydroxymethylated genes in the group of large fish.

Source	Term name	Term ID	p.val.adj (q)
GO:BP	developmental process	GO:0032502	3.60E-12
GO:BP	anatomical structure development	GO:0048856	5.08E-11
GO:BP	multicellular organism development	GO:0007275	1.79E-10
GO:BP	anatomical structure morphogenesis	GO:0009653	2.17E-10
GO:BP	system development	GO:0048731	1.62E-09
GO:BP	tissue development	GO:0009888	5.73E-08
GO:BP	regulation of signaling	GO:0023051	1.23E-07
GO:BP	cellular developmental process	GO:0048869	1.23E-07
GO:BP	regulation of cell communication	GO:0010646	1.26E-07
GO:BP	cell differentiation	GO:0030154	1.31E-07
GO:BP	cell surface receptor signaling pathway	GO:0007166	1.31E-07
GO:BP	regulation of signal transduction	GO:0009966	1.92E-07
GO:BP	multicellular organismal process	GO:0032501	2.76E-07
GO:BP	intracellular signal transduction	GO:0035556	2.76E-07
GO:BP	animal organ development	GO:0048513	2.42E-06
GO:BP	regulation of intracellular signal transduction	GO:1902531	7.49E-06
GO:BP	regulation of response to stimulus	GO:0048583	1.09E-05
GO:BP	regulation of biological process	GO:0050789	2.11E-05
GO:BP	biological regulation	GO:0065007	2.27E-05
GO:BP	regulation of cellular process	GO:0050794	2.46E-05
GO:BP	small GTPase mediated signal transduction	GO:0007264	2.62E-05
GO:BP	chordate embryonic development	GO:0043009	3.95E-05
GO:BP	embryo development ending in birth or egg hatching	GO:0009792	4.13E-05
GO:BP	anatomical structure formation involved in morphogenesis	GO:0048646	4.14E-05
GO:BP	embryo development	GO:0009790	7.37E-05
GO:BP	cell development	GO:0048468	8.32E-05
GO:BP	skeletal system development	GO:0001501	1.15E-04
GO:BP	nervous system development	GO:0007399	1.77E-04
GO:BP	locomotion	GO:0040011	2.51E-04
GO:BP	regulation of small GTPase mediated signal transduction	GO:0051056	2.51E-04
GO:BP	regulation of macromolecule metabolic process	GO:0060255	2.51E-04
GO:BP	signal transduction	GO:0007165	3.66E-04
GO:BP	cell migration	GO:0016477	3.72E-04
GO:BP	signaling	GO:0023052	4.48E-04
GO:BP	regulation of metabolic process	GO:0019222	5.52E-04
GO:BP	Rho protein signal transduction	GO:0007266	5.81E-04
GO:BP	epithelium development	GO:0060429	6.74E-04
GO:BP	regulation of nitrogen compound metabolic process	GO:0051171	6.81E-04
GO:BP	regulation of primary metabolic process	GO:0080090	8.46E-04
GO:BP	enzyme linked receptor protein signaling pathway	GO:0007167	8.59E-04
GO:BP	circulatory system development	GO:0072359	8.59E-04

GO:BP	axon development	GO:0061564	8.70E-04
GO:BP	regulation of cellular metabolic process	GO:0031323	8.70E-04
GO:BP	regulation of gene expression	GO:0010468	8.98E-04
GO:BP	cell communication	GO:0007154	1.00E-03
GO:BP	cellular response to stimulus	GO:0051716	1.08E-03
GO:BP	developmental growth	GO:0048589	1.08E-03
GO:BP	cell motility	GO:0048870	1.14E-03
GO:BP	localization of cell	GO:0051674	1.14E-03
GO:BP	tube development	GO:0035295	1.28E-03
GO:BP	neuron differentiation	GO:0030182	1.50E-03
GO:BP	neuron development	GO:0048666	1.50E-03
GO:BP	generation of neurons	GO:0048699	1.50E-03
GO:BP	regulation of RNA metabolic process	GO:0051252	1.56E-03
GO:BP	movement of cell or subcellular component	GO:0006928	1.58E-03
GO:BP	positive regulation of catalytic activity	GO:0043085	1.64E-03
GO:BP	neurogenesis	GO:0022008	1.68E-03
GO:BP	axonogenesis	GO:0007409	1.85E-03
GO:BP	cellular component morphogenesis	GO:0032989	1.85E-03
GO:BP	transmembrane receptor protein tyrosine kinase signaling pathway	GO:0007169	1.85E-03
GO:BP	cranial nerve development	GO:0021545	1.85E-03
GO:BP	positive regulation of GTPase activity	GO:0043547	1.85E-03
GO:BP	regulation of nucleobase-containing compound metabolic process	GO:0019219	1.90E-03
GO:BP	axon guidance	GO:0007411	1.95E-03
GO:BP	cell part morphogenesis	GO:0032990	1.97E-03
GO:BP	positive regulation of molecular function	GO:0044093	2.00E-03
GO:BP	cell morphogenesis involved in differentiation	GO:0000904	2.06E-03
GO:BP	Ras protein signal transduction	GO:0007265	2.06E-03
GO:BP	neuron projection guidance	GO:0097485	2.08E-03
GO:BP	growth	GO:0040007	2.11E-03
GO:BP	cell morphogenesis involved in neuron differentiation	GO:0048667	2.17E-03
GO:BP	cranial skeletal system development	GO:1904888	2.25E-03
GO:BP	positive regulation of hydrolase activity	GO:0051345	2.25E-03
GO:BP	muscle structure development	GO:0061061	2.36E-03
GO:BP	regulation of GTPase activity	GO:0043087	2.42E-03
GO:BP	response to stimulus	GO:0050896	2.56E-03
GO:BP	cell morphogenesis	GO:0000902	2.65E-03
GO:BP	embryonic viscerocranium morphogenesis	GO:0048703	2.70E-03
GO:BP	regulation of Rho protein signal transduction	GO:0035023	2.79E-03
GO:BP	plasma membrane bounded cell projection morphogenesis	GO:0120039	2.85E-03
GO:BP	neuron projection morphogenesis	GO:0048812	2.85E-03
GO:BP	regulation of Ras protein signal transduction	GO:0046578	2.85E-03
GO:BP	embryonic morphogenesis	GO:0048598	2.85E-03
GO:BP	animal organ morphogenesis	GO:0009887	2.85E-03
GO:BP	cell projection morphogenesis	GO:0048858	2.85E-03
GO:BP	peptidyl-tyrosine phosphorylation	GO:0018108	2.87E-03
GO:BP	protein modification process	GO:0036211	2.93E-03

GO:BP	cellular protein modification process	GO:0006464	2.93E-03
GO:BP	striated muscle tissue development	GO:0014706	3.34E-03
GO:BP	developmental pigmentation	GO:0048066	3.44E-03
GO:BP	peptidyl-tyrosine modification	GO:0018212	3.44E-03
GO:BP	muscle tissue development	GO:0060537	3.60E-03
GO:BP	embryonic skeletal system morphogenesis	GO:0048704	3.65E-03
GO:BP	embryonic cranial skeleton morphogenesis	GO:0048701	3.65E-03
GO:BP	regulation of transcription, DNA-templated	GO:0006355	3.65E-03
GO:BP	central nervous system development	GO:0007417	3.79E-03
GO:BP	skeletal system morphogenesis	GO:0048705	3.79E-03
GO:BP	regulation of nucleic acid-templated transcription	GO:1903506	4.13E-03
GO:BP	regulation of RNA biosynthetic process	GO:2001141	4.13E-03
GO:BP	regulation of cellular macromolecule biosynthetic process	GO:2000112	4.13E-03
GO:BP	neuron projection development	GO:0031175	4.47E-03
GO:BP	biological adhesion	GO:0022610	4.72E-03
GO:BP	cell adhesion	GO:0007155	4.72E-03
GO:BP	nerve development	GO:0021675	4.72E-03
GO:BP	macromolecule modification	GO:0043412	4.95E-03
GO:BP	regulation of macromolecule biosynthetic process	GO:0010556	5.19E-03
GO:BP	regulation of cellular biosynthetic process	GO:0031326	5.43E-03
GO:BP	canonical Wnt signaling pathway	GO:0060070	5.90E-03
GO:BP	regulation of biosynthetic process	GO:0009889	6.29E-03
GO:BP	protein phosphorylation	GO:0006468	6.30E-03
GO:BP	striated muscle cell differentiation	GO:0051146	7.98E-03
GO:BP	aromatic compound biosynthetic process	GO:0019438	8.44E-03
GO:BP	cell migration involved in gastrulation	GO:0042074	9.02E-03

Supplemental Table S2B. Lists of significantly enriched biological processes (BP) for **(B)** hypo-hydroxymethylated genes in the group of large fish.

Source	Term name	Term ID	p.val.adj (q)
GO:BP	cell adhesion	GO:0007155	5.93E-03
GO:BP	biological adhesion	GO:0022610	5.93E-03
GO:BP	cell part morphogenesis	GO:0032990	5.93E-03
GO:BP	cell morphogenesis involved in neuron differentiation	GO:0048667	5.93E-03
GO:BP	axon extension	GO:0048675	5.93E-03
GO:BP	neuron projection morphogenesis	GO:0048812	5.93E-03
GO:BP	cell projection morphogenesis	GO:0048858	5.93E-03
GO:BP	plasma membrane bounded cell projection morphogenesis	GO:0120039	5.93E-03
GO:BP	neuron projection extension	GO:1990138	5.93E-03
GO:BP	response to metal ion	GO:0010038	7.09E-03
GO:BP	cell morphogenesis involved in differentiation	GO:0000904	7.16E-03
GO:BP	axonogenesis	GO:0007409	9.13E-03
GO:BP	response to inorganic substance	GO:0010035	9.13E-03
GO:BP	response to chemical	GO:0042221	9.13E-03

Supplemental Table S2C. Lists of significantly enriched biological pathways (KEGG) for **(C)** hypo-hydroxymethylated genes in the group of large fish.

Source	Term name	Term ID	p.val.adj (q)
KEGG	ECM-receptor interaction	KEGG:04512	2.85E-02
KEGG	Axon guidance	KEGG:04360	2.92E-02

Supplemental Table S2D. Lists of significantly enriched biological pathways (KEGG) for **(D)** hyper-hydroxymethylated genes in the group of large fish.

Source	Term name	Term ID	p.val.adj (q)
KEGG	Axon guidance	KEGG:04360	2.41E-05
KEGG	Focal adhesion	KEGG:04510	2.76E-05
KEGG	ECM-receptor interaction	KEGG:04512	1.33E-04
KEGG	Protein digestion and absorption	KEGG:04974	1.33E-04
KEGG	PI3K-Akt signaling pathway	KEGG:04151	3.91E-04
KEGG	Rap1 signaling pathway	KEGG:04015	2.21E-03
KEGG	Glutamatergic synapse	KEGG:04724	4.99E-03
KEGG	Regulation of actin cytoskeleton	KEGG:04810	4.99E-03

Supplemental Table S3.

A) Total number of DhmCs within gene bodies as resulted from the statistical package limma in R and the annotation using the NCBI *Oreochromis niloticus* annotation release 104. The table shows the ***Chromosome***, ***location*** and ***strand*** information of each DhmC, positive and negative log(fold-change) reflect hyper- and hypo-DhmCs, respectively [**Log(FC)**], adjusted p values calculated with the use of Benjamini-Hochberg FDR correction reflecting the degree of significance between 5hmC levels of the two phenotypes (**adj.P.Val**), gene annotated features of DhmCs based on *Locus* and *Chr* columns (**Annotation**) and their respective ***Reported*** (intron/exon) out of the ***Total*** (introns/exons). ***TranscriptID***, ***GeneID***, ***Gene symbol (Oniloticus RefSeq)***, ***Gene Description*** and ***Transcript Variant*** are reported based on the NCBI *Oreochromis niloticus* annotation release 104 while the modified gene list is based on NCBI and UniProtKB databases [**Gene symbols (NCBI – UniProtKB)**]. **B)** Total number of DhmCs located in intergenic regions. The annotation has been performed based on the closest TSS (**Distance to TSS**) and the gene symbols have been annotated based on the (**Nearest Promoter ID**). **C)** Similarly to **A)** and **B)**, total number of DhmCs occurring within uncharacterized predicted proteins.

Supplemental_Table_S3.xlsx

Supplemental Table S4. List of growth-related genes from functional enriched term “GO:0040007 - growth” (q<0.01), containing differentially hydroxymethylated cytosines (DhmCs) between the two phenotypes (n=5). Columns reflect the chromosomes (**Chr**), the location of the reported DhmC (**Locus**), strand information (**Strand**), positive and negative log(fold-change) reflect hyper- and hypo-DhmCs respectively [**Log(FC)**], adjusted p values calculated with the use of Benjamini-Hochberg FDR correction reflecting the degree of significance between 5hmC levels of the two phenotypes (**adj.P.Val**), gene annotated features of DhmCs based on *Locus* and *Chr* columns. For introns and exons the (reported/total) number of introns and exons is shown and for intergenic DhmCs the distance to the closest TSS (**Annotation**), gene symbols based on the *Oreochromis niloticus* NCBI Annotation Release 104 (**Gene symbols**), hyperlinks to the NCBI protein database based on *Locus* and *Chr* columns (**Protein**), function of the protein (**Function**) based on the provided literature (**References**).

Supplemental_Table_S4.xlsx

Supplemental Table S5. Columns reflect the chromosome (**CHR**), the reported single nucleotide polymorphism (**SNP**), the location of the reported SNP (**BP**), the identified polymorphism/-s in columns (**A1**) and (**A2**), the frequency of A1 polymorphism/-s in the small-sized (**FreqA1_Small**) and the frequency of A1 polymorphism in the large-sized (**FreqA1_Large**) fish group as well as the p value reflecting the degree of sigificance. **A)** List of SNPs as resulted from software Stacks after filtering for missing values. **B)** List of SNPs with a p value <0.01. **C)**

Supplemental_Table_S5.xlsx

Supplemental Figures

Supplemental Figure S1

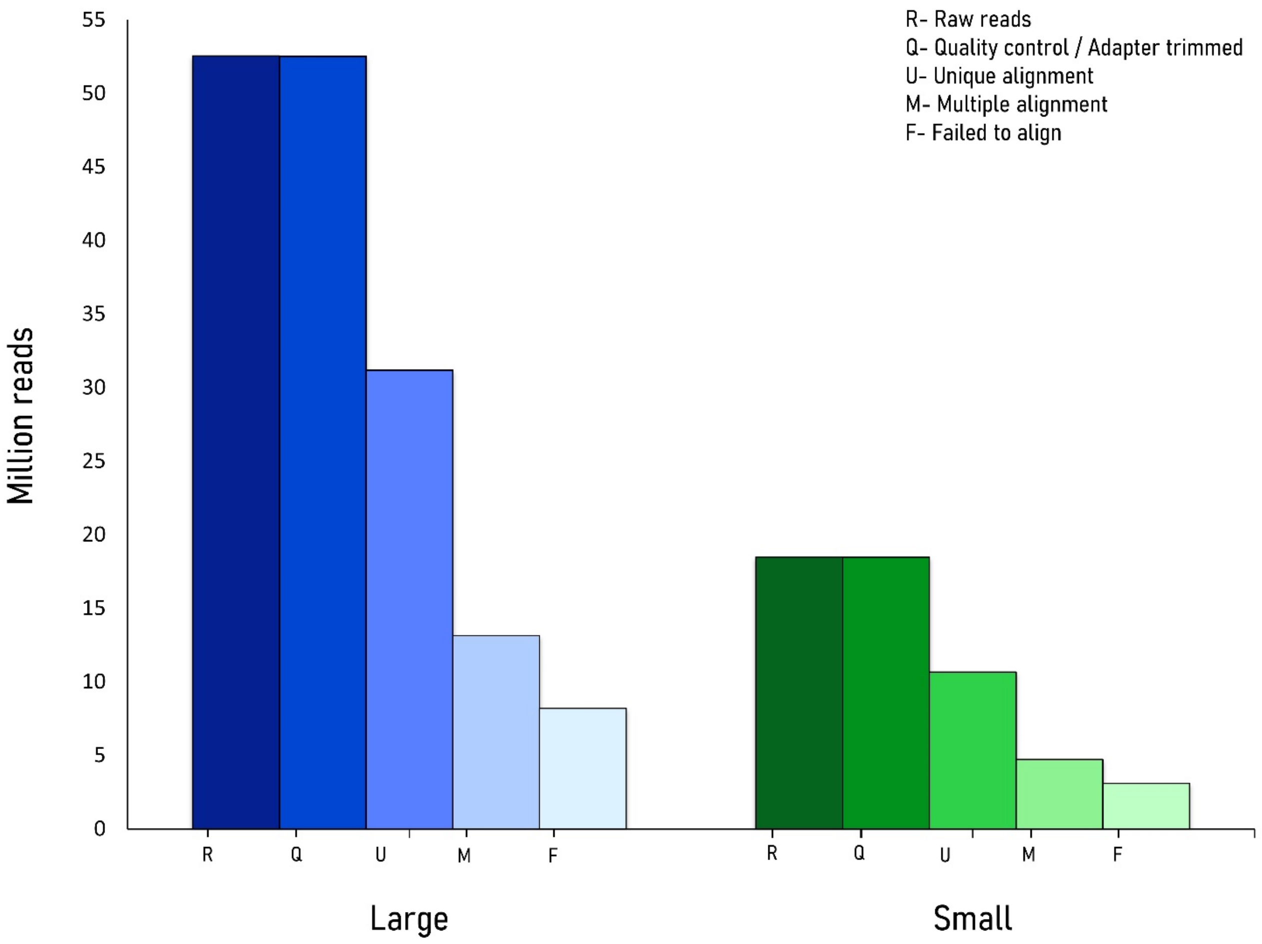

Supplemental Fig. S1. Barplot of the average number of total raw (R), quality/adapter-trimmed (Q), uniquely mapped (U), multiple mapped (M) and failed to map (F) sequencing reads as depicted from the RRHP libraries representing the large (blue) and small (green) groups of fish, respectively(n=5).

Supplemental Figure S2

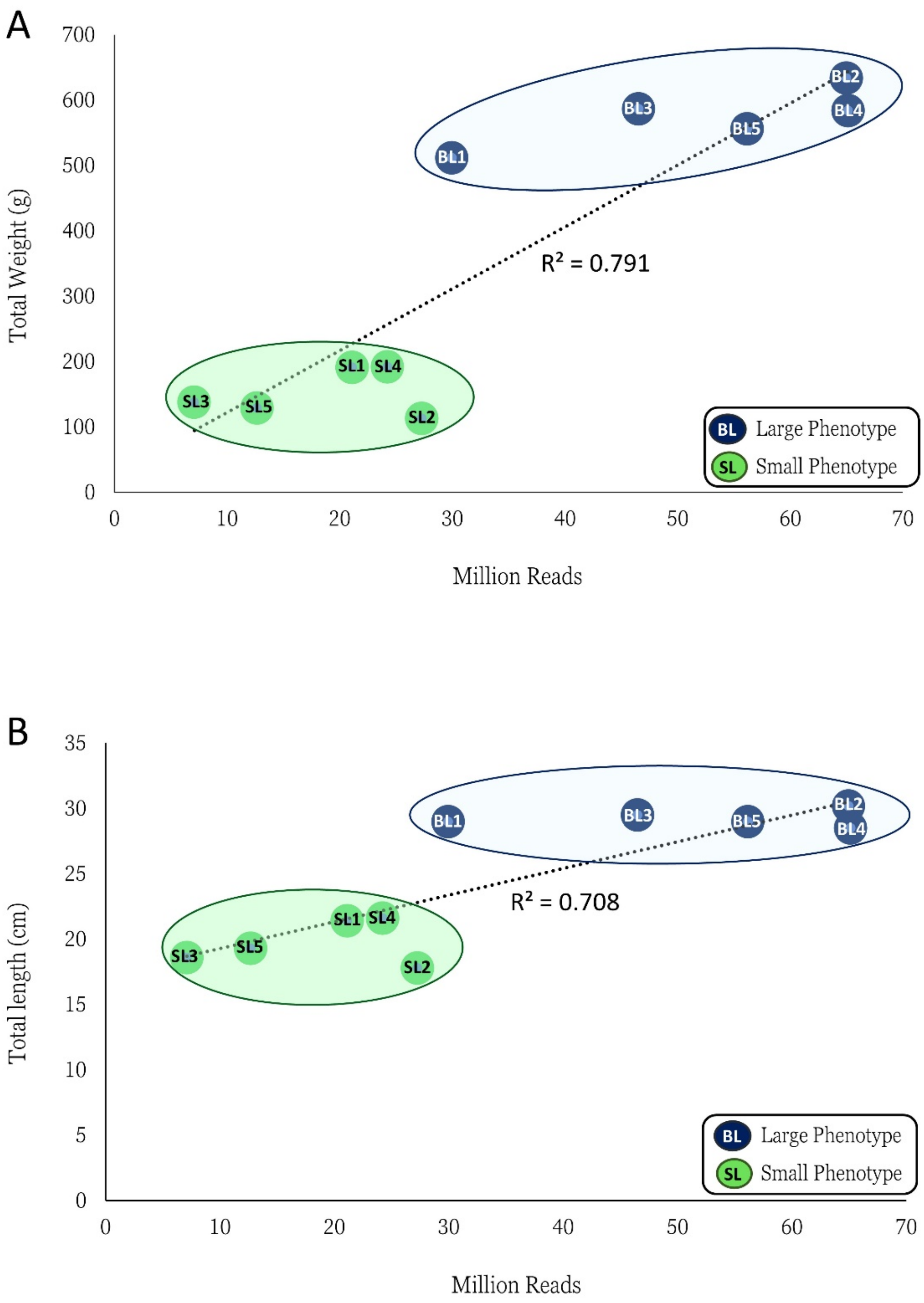

Supplemental Fig. S2. Scatter plot representing the correlation of weight (**A**) and length (**B**) with the number of raw sequencing reads. In both plots, the samples BL1-5 (dark blue) and SL1-5 (green) represent the 5 large and 5 small individuals, respectively (n=10). The y-axis corresponds to the weight (**A**) and length (**B**) measurements, while the x-axis corresponds to the raw number of sequencing reads in millions. Pearson's correlation coefficient, R^2 and p-value were calculated for both plots. (**A** - ρ=0.889, R^2=0.791, p.val=0.0005; **B** - ρ=0.842, R^2=0.708, p.val=0.0023).

Supplemental Figure S3

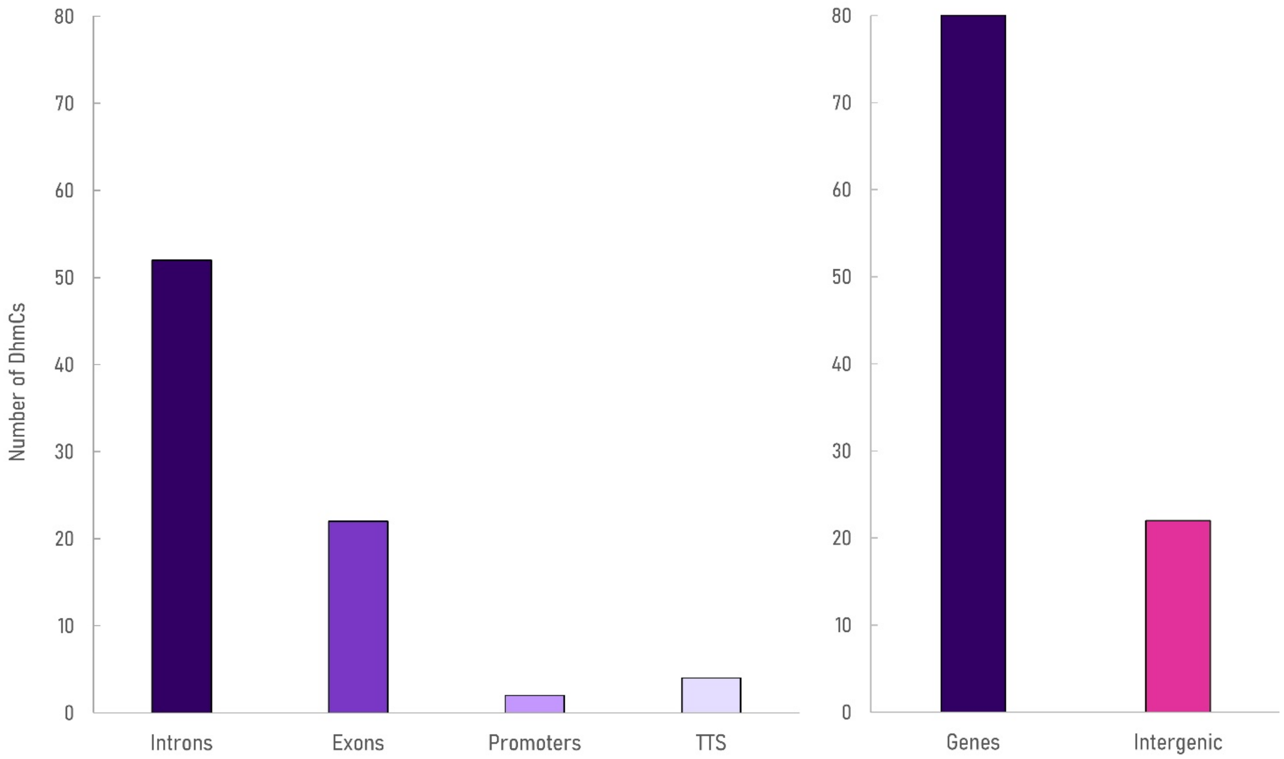

Supplemental Fig. S3. Barplot representing the enrichment of DhmCs within the annotated features of growth-related genes. The x-axis shows gene body-related annotated features in different shades of purple (introns, exons, promoters and transcription termination sites-TTS). The bar "Genes" (dark purple) is a stacked bar of all the DhmCs within gene bodies, promoters and TTS, except the DhmCs within intergenic regions (pink bar). The y-axis represents the number of DhmCs that correspond to the above features as shown in x-axis.

Supplemental Figure S4

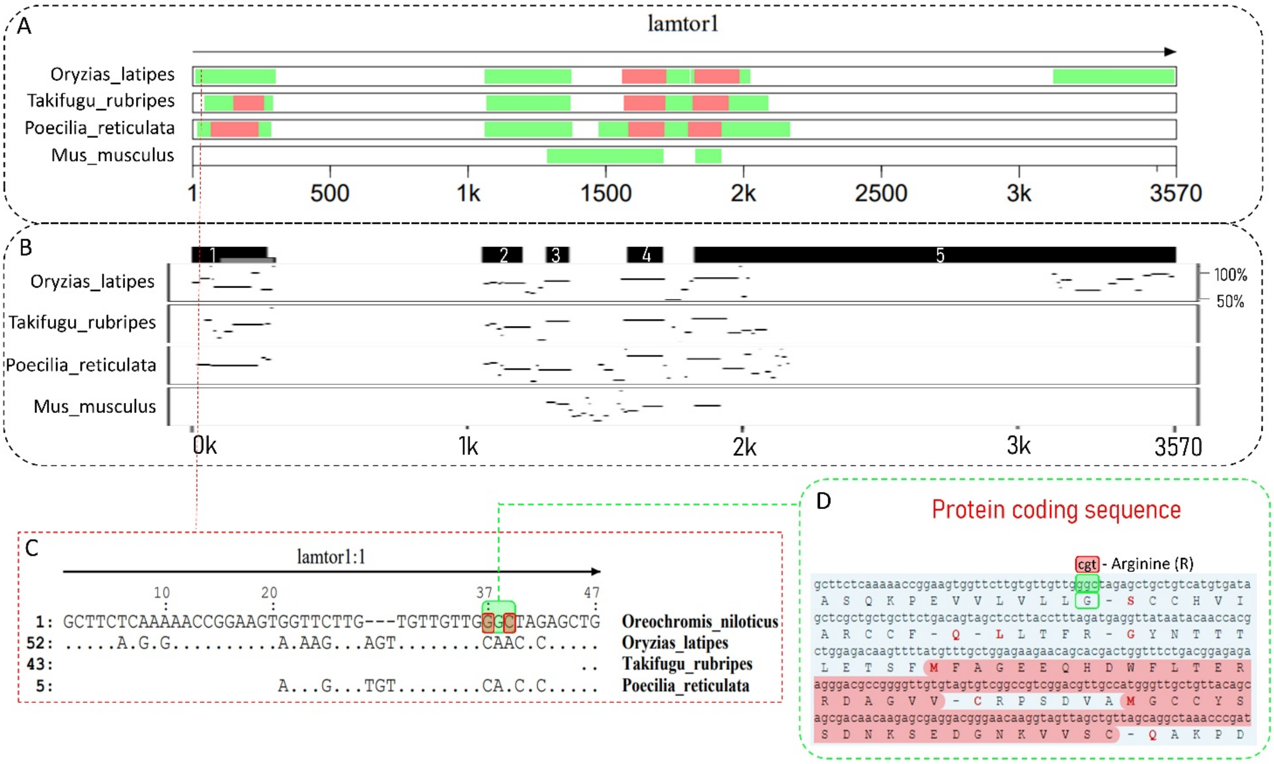

Supplemental Fig. S4. A) Visual representation of conserved regions of *lamtor1* among four fish species and the mouse (*Mus musculus*). Highly conserved, intermediately conserved and non-conserved regions of the gene are represented by red, green and white colour, respectively. **B**) Numbered black boxes at the top represent the exons of *lamtor1* in Nile tilapia while each dot-plot corresponds to the pairwise alignments (50-100% similarity) between Nile tilapia and the species that are designated on the left side. **C**) The exact position and sequence of the significant SNPs ($p<0.01$) are shown in red colour while the codon is marked in green. **D**)The promoter and protein coding sequence of *lamtor1*. The reference codon and amino acid are marked with green while their substitutions are marked with red colour.
